# 1157. The Clinical Impact of Casirivimab–Imdevimab Monoclonal Antibody Therapy (CIMAT) in COVID-19 patients: A real-world insight from India.

**DOI:** 10.1093/ofid/ofac492.995

**Published:** 2022-12-15

**Authors:** Jose J Kochuparambil, Aleena Issac

**Affiliations:** St. Joseph's College of Pharmacy, Cherthala, Pala, Kerala, India; St. Joseph's College of Pharmacy, Pathanamthitta, Kerala, India

## Abstract

**Background:**

Various clinical studies from developed countries support the use of Casirivimab–Imdevimab Monoclonal Antibody Therapy (CIMAT) to treat outpatients with mild to moderate coronavirus disease-19 (COVID-19). However, the real-world clinical data from India, owing to the portrayed benefits, are limited. Moreover, the choice of CIMAT depends upon the willingness of the patient or his bystanders for this therapy. The study aimed to assess the impact of Casirivimab–Imdevimab Monoclonal Antibody Therapy (CIMAT) for mild to moderate COVID-19 in India.

**Methods:**

A single-center, retrospective comparative study was conducted to evaluate the impact of the CIMAT in symptomatic adult COVID-19 patients admitted to a secondary care hospital in South India, categorized as mild or moderate, and who are at high risk for progression to severe COVID-19. The data for the study were retrieved from the Electronic Health Records (EHR) of the hospital from April 2021 to March 2022, a period of 12 months. The primary outcome of the study was the length of hospitalization, and the secondary outcomes were mechanical ventilation post-infusion, readmissions after discharge, and mortality rate. Outcomes in the CIMAT cohort were compared to those of a control group of patients admitted with a diagnosis of SARS-CoV-2 during the same period who were qualified for CIMAT but were unwilling to administer it.

**Results:**

This study included 474 patients, 48 in the CIMAT cohort and 426 in the control group. In the CIMAT cohort, the median age was 65 years (IQR: 50–73); 56.2% were ≥65 years old; 54.3% were males. Patients on CIMAT compared to the control group had a lower length of hospitalization(median: 4 [IQR 1–8]), lesser requisite for mechanical ventilation (4.3% vs 20.8%, p < 0.001) and less frequent readmissions within 10 days post-discharge (6.5% vs 9.3%, p < 0.001). Mortality was more among the control group, i.e., 22 (5.1%), and only one patient in the CIMAT cohort died during hospitalization (p = 0.23). The majority of the patients in the CIMAT cohort (41[89.1%]) were symptom-free within seven days of antibody cocktail administration.

**Conclusion:**

Patients treated with CIMAT for COVID-19 were more clinically benefitted than those treated without the antibody cocktail.

**Disclosures:**

**All Authors**: No reported disclosures.

